# Epidemiological characteristics of single and co-infections with upper respiratory tract pathogens among children in eastern China: a retrospective study

**DOI:** 10.3389/fped.2026.1694989

**Published:** 2026-03-18

**Authors:** Ruizhe Yang, Xianglong Meng, Nannan Wang, Yangyang Zhao, Jing Zhou, Zhong Zhang

**Affiliations:** Department of Public Health, Children’s Hospital of Nanjing Medical University, Nanjing, China

**Keywords:** children, co-infection, eastern China, epidemiological characteristics, upper respiratory tract pathogens

## Abstract

**Background:**

The epidemiology of pediatric respiratory tract infections (RTIs) has shifted since the COVID-19 pandemic. This study aimed to characterize the prevalence and co-infection patterns of seven key respiratory pathogens in children in Eastern China following the relaxation of pandemic-related non-pharmaceutical interventions.

**Methods:**

We conducted a large-scale, retrospective study involving pediatric patients aged under 18 years who presented with symptoms of acute respiratory tract infection (RTI) to a tertiary academic children's hospital between April 2,023 and March 2,024. Nasopharyngeal or oropharyngeal swab specimens were collected and tested using multiplex reverse transcription polymerase chain reaction (RT-PCR) for seven common respiratory pathogens: *Mycoplasma pneumoniae (M. pneumoniae)*, adenovirus (ADV), influenza A virus (IAV), influenza B virus (IBV), human rhinovirus (HRV), respiratory syncytial virus (RSV), and SARS-CoV-2. Stratified analyses were performed across four predefined age groups.

**Results:**

Pathogen distribution showed distinct age-related patterns. Among high-risk pathogens, infants with RSV infection were the most common. Conversely, IAV and *M. pneumoniae* prevalence rose with age. Infants with IAV infection increased from 1,925 (9.1%) to 18,930 (17.5%) in school-age children with IAV infection, while Infants with *M. pneumoniae* infection surged from 2,812 (13.3%) to a peak of 48,284 (44.6%) in the oldest group with *M. pneumoniae* infection. For lower-risk pathogens, Children with ADV infection and those with IBV infection also increased steadily with age, reaching 20,661 (19.1%) and 11,574 (10.7%) respectively in school-age children. Toddlers with HRV infection peaked at 7,501 (14.0%), while Infants with SARS-CoV-2 infection were the most common (5,244, 24.8%), followed by school-age children with SARS-CoV-2 infection (12,331, 11.4%). Co-infections were significant, with children with *M. pneumoniae* infection being the most likely to have co-infections—frequently paired with HRV infection (2,143 children), ADV infection (1,100 children), and IAV infection (1,063 children).

**Discussion:**

The distinct epidemiological patterns and high rates of co-infection highlight the need for age-specific prevention strategies. The significant role of *M. pneumoniae* in both single and co-infections underscores the clinical utility of multiplex diagnostics to guide appropriate antimicrobial stewardship.

**Conclusion:**

This study reveals significant age-dependent variations and high co-infection rates for seven respiratory pathogens in a large pediatric cohort. These findings are crucial for informing public health policies and optimizing the clinical management of pediatric RTIs in the post-pandemic era.

## 1 Background

Children and adolescents with Respiratory tract infections (RTIs) are the leading reason for medical consultations in pediatric settings, with viral pathogens being the predominant etiological agents during childhood Zhao, Ke (1). Studies indicate that respiratory pathogens account for up to 90% of lower RTIs in infants (2). Notably, Younger children with symptomatic episodes show higher frequencies of positive test results, emphasizing the substantial influence of age on the clinical presentation of viral infections (3). Recent evidence indicates that the epidemiology of pediatric respiratory pathogens has undergone substantial shifts following the relaxation of non-pharmaceutical interventions (NPIs) implemented during the COVID-19 pandemic. Multiple countries have reported atypical seasonal resurgences, increased incidence rates, and altered age distributions for common respiratory viruses, including influenza, RSV, and rhinovirus (4, 5). These changes are thought to result from reduced population-level immunity due to prolonged suppression of viral circulation, a phenomenon often referred to as “immunity debt” Cohen, Ashman (6).

Co-infections represent an additional and increasingly recognized challenge in pediatric RTIs. Molecular surveillance studies suggest that viral–viral and viral–bacterial co-infections may exacerbate disease severity, prolong symptom duration, and complicate clinical management, particularly among infants and school-aged children (7, 8). *M. pneumoniae*, in particular, has been frequently identified in mixed infections with respiratory viruses, raising concerns regarding delayed diagnosis and inappropriate antimicrobial use (9). Understanding age-specific susceptibility patterns and seasonal co-infection dynamics is therefore critical for optimizing diagnostic strategies, guiding antimicrobial stewardship, and informing targeted public health interventions.

A critical challenge arising from the high incidence of pediatric RTIs is the difficulty in clinically distinguishing between viral and bacterial etiologies, which often present with overlapping symptoms (10, 11). This diagnostic uncertainty frequently leads to the excessive use of antibiotics for what are essentially self-limiting viral infections (12, 13). Such inappropriate prescribing is a primary driver of antimicrobial resistance (AMR), a silent pandemic that threatens to undermine modern medicine (14). With the global burden of AMR having caused an estimated 1.27 million deaths in 2019 and projected to cause 10 million deaths annually by 2050 if no urgent action is taken, strategies that promote judicious antibiotic use are of paramount importance (15, 16). Therefore, understanding the precise epidemiological landscape of respiratory pathogens is not merely an academic exercise, it is a fundamental prerequisite for effective antimicrobial stewardship, enabling clinicians to make evidence-based treatment decisions that preserve the efficacy of life-saving antibiotics (17).

In the aftermath of the COVID-19 pandemic, viral nucleic acid detection technology has not only facilitated more accurate clinical prognostic assessments but also enabled healthcare facilities to implement robust infection prevention and control strategies during outbreaks. This approach effectively disrupts the transmission chain, thereby minimizing viral spread and advancing diagnostic and therapeutic methodologies in meaningful ways (18). Rapid pathogen identification is key to effective treatment in critical areas. For example, children with respiratory infections often exhibit similar symptoms (e.g., cough, sore throat, fever) caused by various pathogens (2). Precise identification of the causative agent reduces unnecessary antibiotic use, avoids ineffective tests for viral infections, eases healthcare system burdens, and enables optimal treatment strategies (19, 20). Public health authorities use epidemiological data, including information on emerging virus strains, to refine prevention and control strategies during outbreaks. Pathogen nucleic acid testing provides reliable data to support these efforts (1). Advancements in multiplex PCR for rapid virus diagnosis and innovations in bacterial infection identification have enabled research on co-infection interactions and their impacts on host health (3).

The Children's Hospital of Nanjing Medical University, a leading children's hospital in East China, serves Jiangsu, Anhui, and other provinces. Since 2023, it has implemented multi-pathogen testing for pediatric RTIs to improve diagnosis, clinical management, and regional epidemiological research. This study analyzed the epidemiological characteristics of single and mixed upper respiratory infections using multiplex PCR data from the 2023–2024 season, after COVID-19 restrictions eased. Our results emphasize the importance of ongoing respiratory pathogen monitoring for evidence-based strategies to prevent and control pediatric RTIs in East China.

## 2 Materials and methods

### 2.1 Study population and eligibility criteria

Pediatric patients aged <18 years who presented to outpatient clinics or were admitted to inpatient wards between April 2023 and March 2024 with symptoms consistent with upper respiratory tract infection (URTI), including fever, cough, sore throat, nasal congestion, or rhinorrhea, were eligible for inclusion. Patients were excluded if duplicate test records were identified or if essential demographic or laboratory data were incomplete. Age stratification followed commonly applied pediatric epidemiological groupings (<1 year, 1–3 years, 3–6 years, and 6–18 years), which facilitate comparisons across developmental stages and are recommended in respiratory surveillance studies (3).

### 2.2 Specimen collection and handling

Nasopharyngeal or oropharyngeal swab specimens were collected from each patient by trained healthcare personnel in accordance with standardized clinical sampling protocols (21). Only one specimen per patient per visit was included to minimize sampling bias. Specimens were transported promptly to the hospital's certified clinical microbiology laboratory and processed according to manufacturer instructions and laboratory quality control standards. The use of upper respiratory tract specimens for molecular detection is well established and provides high diagnostic yield for viral and atypical bacterial respiratory pathogens in children (22, 23).

### 2.3 Pathogen selection and molecular detection

Seven respiratory pathogens of clinical and public health significance were included: *M. pneumoniae*, adenovirus (ADV), influenza A virus (IAV), influenza B virus (IBV), human rhinovirus (HRV), respiratory syncytial virus (RSV), and SARS-CoV-2. These pathogens were selected due to their high prevalence in pediatric upper respiratory tract infections (URTI) and their well-documented association with seasonal outbreaks and co-infections (24). Nucleic acid extraction and detection were conducted using commercially available real-time polymerase chain reaction (RT-PCR) assays (25). RT-PCR is recognized as the reference standard for respiratory pathogen detection owing to its high sensitivity and specificity, and is widely recommended for both epidemiological surveillance and clinical diagnostics (26, 27). Multiplex PCR platforms allow for the simultaneous detection of multiple pathogens from a single specimen and are particularly valuable in identifying co-infections in pediatric respiratory diseases (28, 29).

### 2.4 Statistical analysis

Descriptive statistics were used to summarize demographic characteristics and pathogen detection rates (30). Categorical variables were expressed as frequencies and percentages. Differences in pathogen positive rates across age groups and months were assessed using the chi-squared (*χ*^2^) test. Statistical significance was defined as a two-sided *p* value < 0.05. Statistical analyses were performed using R software (version 4.3.0). The analytical approach followed standard methods applied in large-scale respiratory pathogen surveillance studies (31).

### 2.5 Ethics approval and consent to participate

This study obtained ethics approval from the Institutional Ethics Committee (IEC) of the Children's Hospital of Nanjing Medical University (Ethics Approval Number: 202410012-1). Given that this was a retrospective study and all participant data were sourced exclusively from pre-exist electronic medical records, we submitted an application for a waiver of informed consent. This request was subsequently approved by the IEC.

## 3 Result

### 3.1 Pathogen distribution across age groups

**Figure 1:**
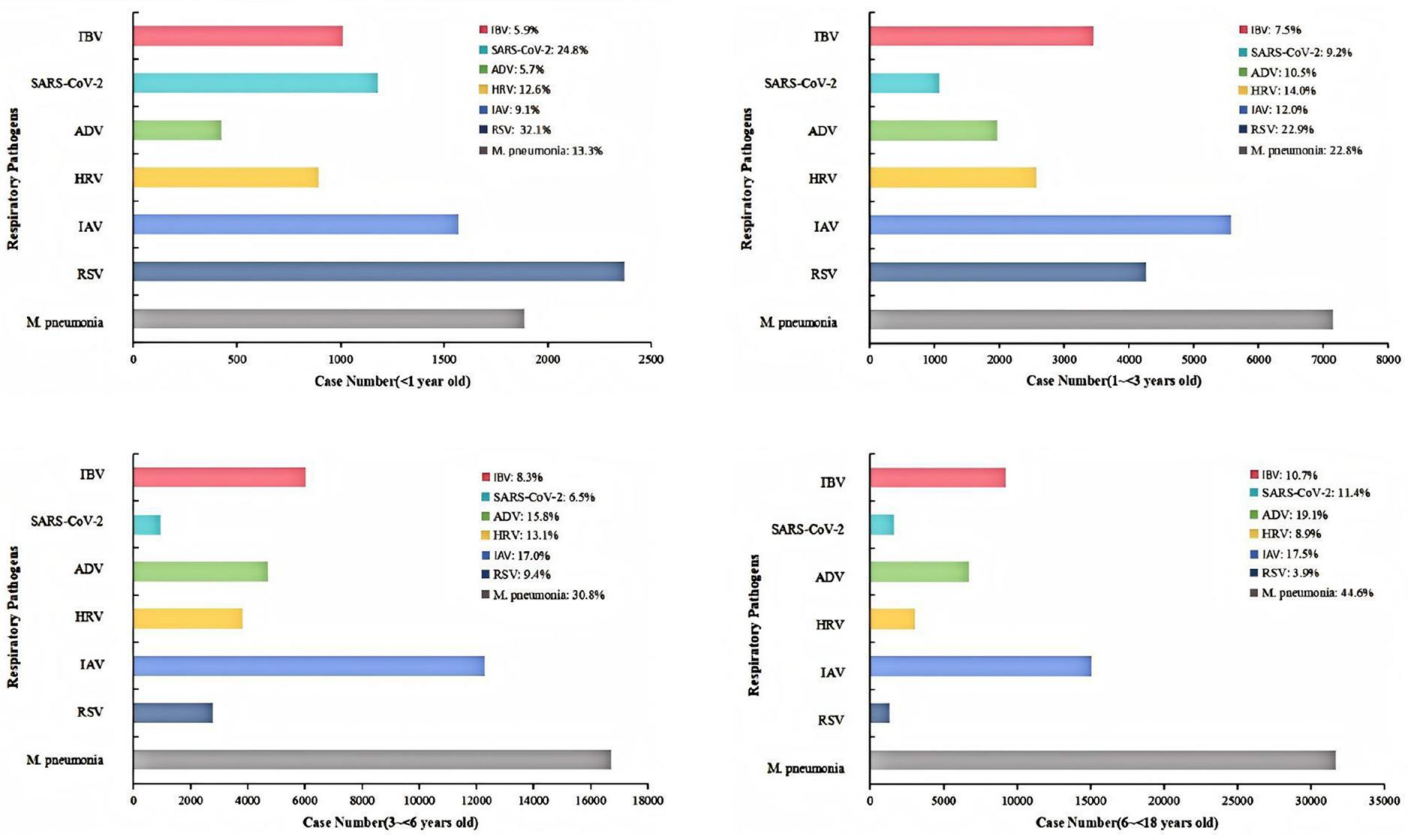
Distribution of respiratory pathogens in children by Age group. This figure displays the prevalence of various respiratory pathogens among children, segmented into four distinct age groups: younger than 1 year, 1 to younger than 3 years, 3 to younger than 6 years, and 6 to younger than 18 years. The data is presented in four corresponding charts, with each chart using a distinct color to represent a specific pathogen and providing the exact percentage of cases attributed to it. The case numbers for each age group are indicated on the *x*-axis, allowing for a clear visual comparison of the pathogen distribution across different stages of childhood.

A total of 270,526 patients were included in this study. The study population was categorized into four distinct age groups: 21,145 infants (<1 year), 53,575 toddlers (1–3 years), 87,635 preschool children (3–6 years), and 108,171 school-age children (6–18 years). Seven respiratory pathogens were identified across four distinct pediatric age groups: <1 year old, 1–<3 years old, 3–<6 years old, and 6–<18 years old. The distribution and prevalence of these pathogens varied significantly across the different age cohorts (Figure 1). HRV infection was the most frequently identified in children aged 1 to <3 years, with 14.0% of tested children in this age group having HRV infection. In children aged <1 year and 3–<6 years, 12.6% and 13.1% of tested children, respectively, had HRV infection. The lowest proportion of children with HRV infection was in those aged 6–<18 years, at 8.9% of tested children.

The highest proportion of children with IAV infection was in those aged 6–<18 years, at 17.5% of tested children. The 3–<6 years old and 1–<3 years old groups showed similar prevalence, with 17.0% and 12.0% of cases, respectively. The lowest proportion of IAV was in the <1 year old group, at 9.1%.

IBV prevalence increased with age. The highest proportion of IBV was observed in the 6–<18 years old group (10.7%), followed by the 3–<6 years old group (8.3%), the 1–<3 years old group (7.5%), and the <1 year old group (5.9%).

ADV infections were most prevalent in the 6–<18 years old group, accounting for 19.1% of cases. The 3–<6 years old age group also showed a high proportion of ADV at 15.8%. The prevalence was lower in the younger age groups, with 10.5% in the 1–<3 years old group and 5.7% in the <1 year old group.

RSV infection was the most dominant in infants <1 year old, with 32.1% of tested infants in this age group having RSV infection. The prevalence decreased sharply with age, with 22.9% of cases in the 1–<3 years old group, 9.4% in the 3–<6 years old group, and the lowest at 3.9% in the 6–<18 years old group.

*M. pneumoniae* infection showed a clear trend of increasing prevalence with age; it was most common in the oldest age group (6–<18 years old), with 44.6% of tested children in this group having *M. pneumoniae* infection. The prevalence was 30.8% in the 3–<6 years old group, 22.8% in the 1–<3 years old group, and 13.3% in the <1 year old group.

The highest proportion of SARS-CoV-2 was found in the <1 year old group, at 24.8%. The prevalence was lower in the other age groups, with 11.4% in the 6–<18 years old group, 9.2% in the 1–<3 years old group, and 6.5% in the 3–<6 years old group.

### 3.2 Co-infection of seven respiratory pathogens in children

An analysis of co-infection rates among patients revealed significant patterns of co-occurrence involving seven respiratory pathogens. *M. pneumoniae*, ADV, IAV, HRV and RSV were central to the most frequent co-infections.

*M. pneumoniae* infection was the most common to co-occur with other pathogens in tested children. The highest co-infection rate was among children with both *M. pneumoniae* and HRV infections, identified in 2,143 children (21.73%). *M. pneumoniae* also showed a high rate of co-infection with ADV, occurring in 1,100 cases, and with IAV in 1,063 cases.

Following the pairings with *M. pneumoniae*, ADV also demonstrated significant co-infection rates with other major viruses. It was most commonly co-detected with HRV in 742 cases (7.52%) and with RSV in 622 cases (6.31%). These findings establish ADV as a key pathogen in the co-infection landscape.

While many pathogen pairs showed high rates of co-detection, some pairings were rare or entirely absent, such as IBV and SARS-CoV-2. This absence of co-occurrence may suggest a competitive interaction between these two specific viruses that could be a subject for future investigation (Table 1).

**Table 1:** Co-infection of seven respiratory pathogens in children.

*M. pneumoniae*	RSV	IAV	HRV	ADV	SARS-CoV-2	IBV
*M. pneumoniae*	744 (7.54%)	1,063 (10.78%)	2,143 (21.73%)	1,100 (11. 15%)	10 (0. 10%)	297 (3.01%)
RSV		118 (1.20%)	702 (7. 12%)	622 (6.31%)	0 (0.00%)	292 (2.96%)
IAV			284 (2.88%)	367 (3.72%)	278 (2.82%)	326 (3.31%)
HRV				742 (7.52%)	4 (0.04%)	162 (1.64%)
ADV					3 (0.03%)	463 (4.69%)
SARS-CoV-2 IBV						143(1.45%)

The positive rate (%) represents the percentage of positive dual co-infection cases out of the total 9,863 co-infection cases.

**Figure 2:**
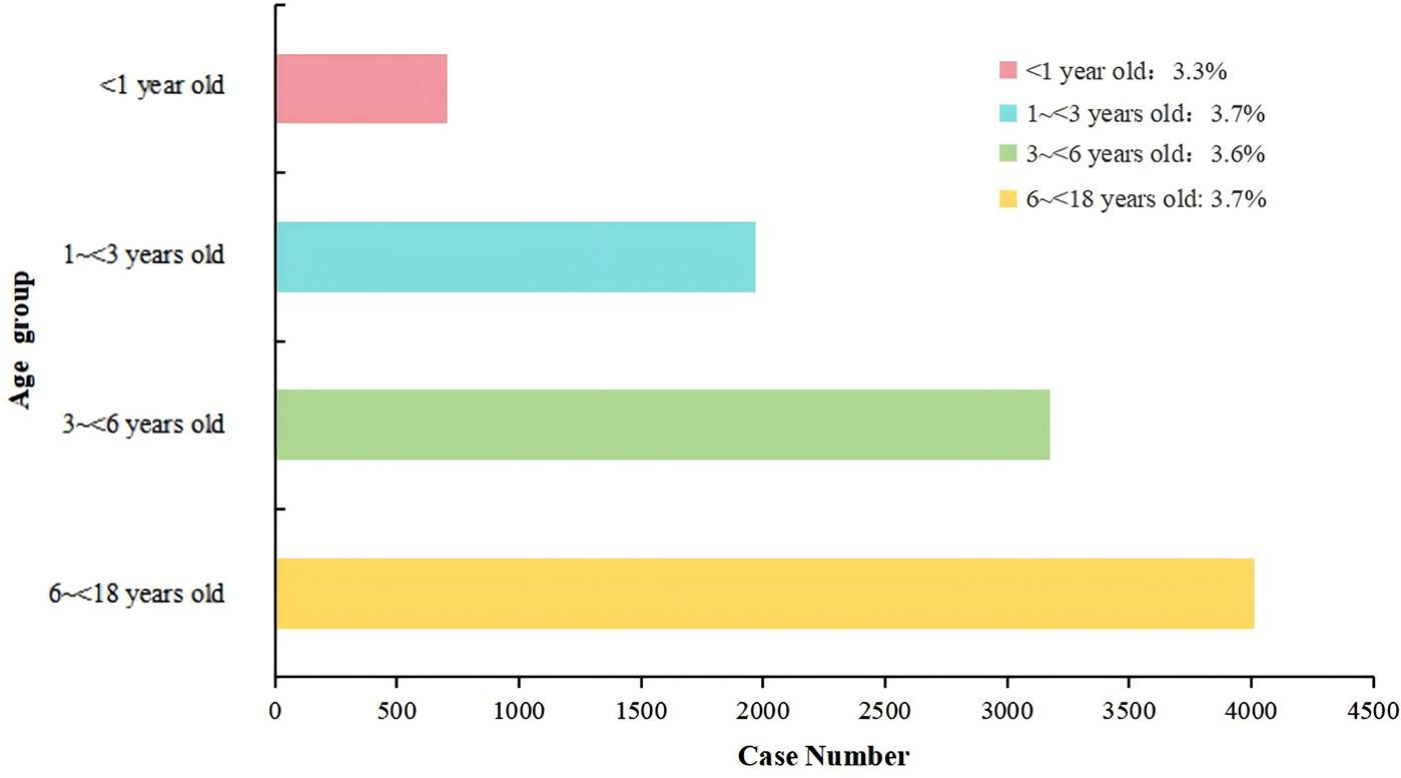
Distribution of Co-infections by pathogen type and Age group. This figure provides a comprehensive analysis of respiratory pathogen co-infections in children, segmented into four age groups: younger than 1 year, 1 to younger than 3 years, 3 to younger than 6 years, and 6 to younger than 18 years. The data is presented in four separate charts, each corresponding to an age group. Within each chart, the distribution of co-infections is broken down by the specific pathogens involved.

The analysis of co-infection distribution across different age groups revealed a relatively uniform pattern. Among the examined cases, children aged 6–<18 years accounted for 3.7% of all co-infected children. Similarly, children aged 1 to less than 3 years old also represented 3.7% of the cases. The age group of 3 to less than 6 years old showed a comparable distribution, contributing to 3.6% of the total cases. The youngest group, infants under 1 year old, comprised 3.3% of the cases. Overall, the distribution of pathogens remained consistent across the four specified age groups, with minimal variation observed (Figure 2).

### 3.3 Monthly positive cases of seven respiratory pathogens

**Figure 3:**
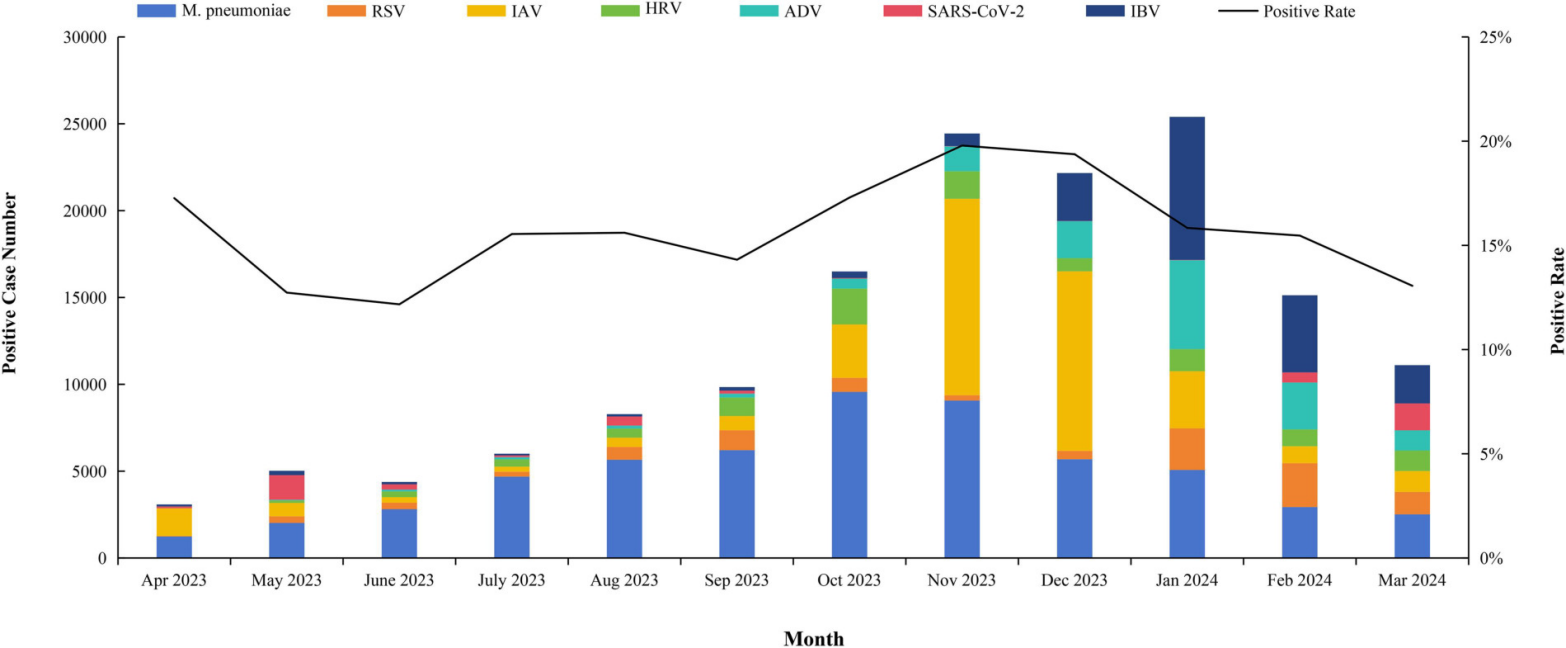
Monthly distribution of positive cases for seven respiratory pathogens. This line graph illustrates the seasonal circulation patterns of seven common respiratory pathogens throughout a calendar year. The *x*-axis represents the months, from January to December, and the *y*-axis quantifies the number of positive cases for each pathogen. Each distinctively colored line tracks the monthly prevalence of a specific agent. The figure clearly demonstrates the varying seasonality of these infections, highlighting peak periods and fluctuations in transmission for each pathogen over the course of the year. The positive rate is the percentage of individuals who tested positive for a specific pathogen out of the total number of individuals who were tested for that pathogen.

From April 2023 to March 2024, the overall rate of children testing positive for respiratory pathogens fluctuated throughout the year, with the highest rates observed in the winter months. The pathogen landscape demonstrated significant monthly variation in composition and proportion. During the spring and early summer of 2023 (April-July), overall detection rates were relatively low, with *M. pneumoniae* infection being the most predominant, while IAV and SARS-CoV-2 infections were highly detected in specific periods among tested children. A shift occurred in the late summer and autumn (August-October), marked by a substantial increase in the proportion of children with IAV infection, while *M. pneumoniae* still was the single most dominant pathogen. The winter months (November 2023–January 2024) exhibited the most dynamic changes. In November, IAV and *M. pneumoniae* began a rapid surge. This trend continued into December, where the proportion of children with IAV infection reached its peak among all detected infections, closely followed by rising IFA rates. By January 2024, the composition shifted dramatically again, with IBV peaking and becoming the co-dominant pathogen alongside ADV, while the proportion of RSV began to wane. As the year concluded in February and March 2024, the rates of IAV and RSV declined sharply, and the pathogen profile started to revert towards the spring baseline, with SARS-CoV-2 once again constituting the majority of the reduced number of positive detection. Other pathogens, including ADV, *M. pneumoniae*, and IBV, were detected at consistently low rates throughout the year, without showing a strong seasonal pattern (Figure 3).

### 3.4 Monthly distribution of seven respiratory pathogens

**Figure 4:**
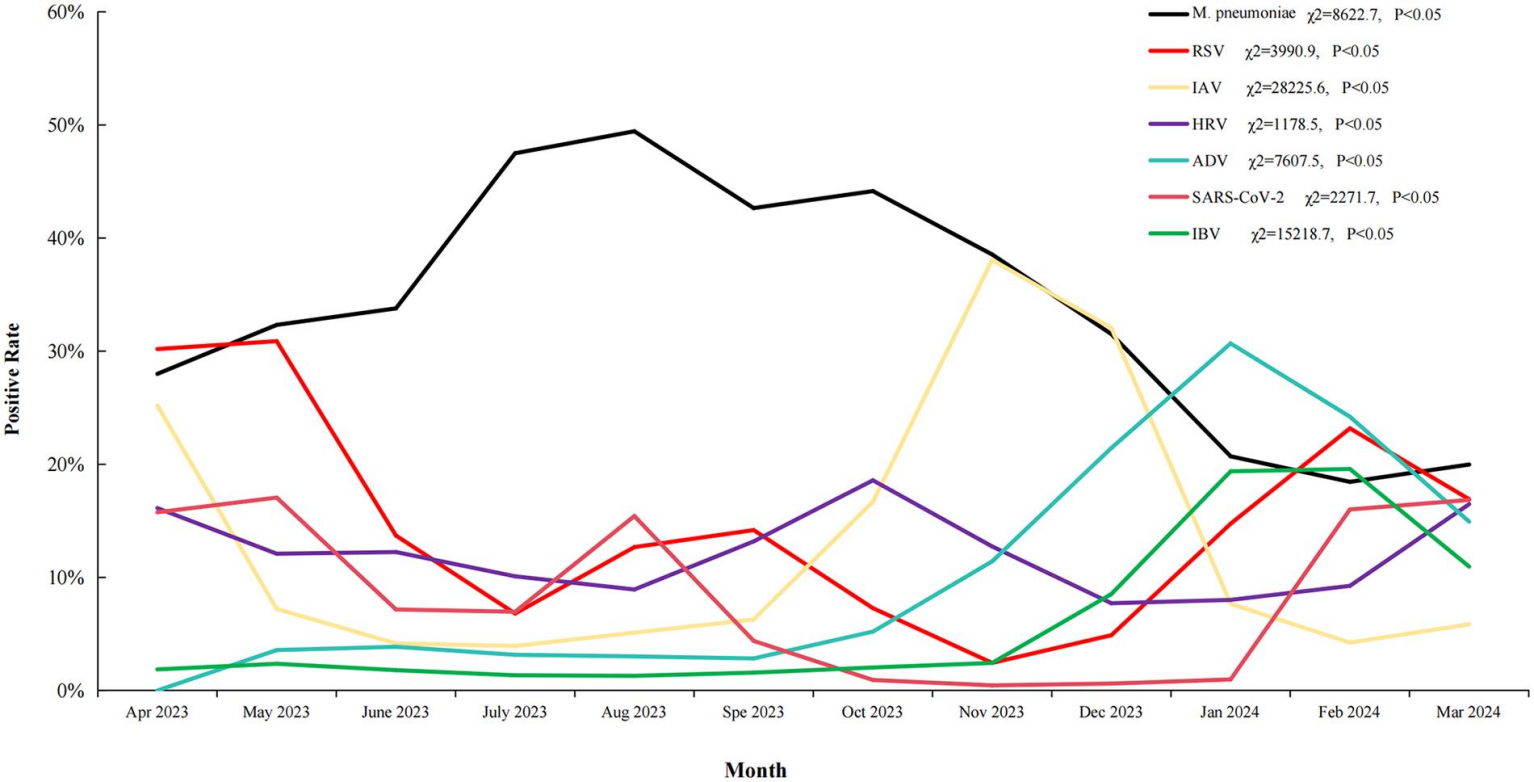
Monthly distribution of respiratory Co-infection cases. This figure displays the seasonal trends of respiratory co-infections throughout a calendar year. The *x*-axis indicates the months from January to December, and the *y*-axis quantifies the number of co-infection cases. Each colored line on the graph represents a specific pairing of co-infecting pathogens. The category is also included to represent all other combinations of co-infections. The graph effectively illustrates the temporal fluctuations and peak seasons for various polymicrobial respiratory infections.

The monthly distribution of the seven respiratory pathogens from April 2023 to March 2024 is presented in Figure 4. Overall, *M. pneumoniae* infection emerged as the most predominant, with a marked increase in children with this infection during the autumn and winter months. In contrast, other pathogens exhibited distinct seasonal patterns. IAV and IBV infections demonstrated heightened activity during the winter season, with peak incidence of children with these infections occurring between December 2023 and February 2024. RSV also displayed a pronounced winter predominance, with the highest detection rates recorded from November 2023 to January 2024. HRV and ADV were detected year-round. HRV showed a moderate increase during the autumn months, whereas ADV exhibited a more prominent peak during the summer and early autumn. SARS-CoV-2 infection persisted throughout the study period, with a notable surge in the number of children testing positive for it during June and July 2023. Statistical analysis revealed significant monthly variations for all seven pathogens (*P* < 0.05), highlighting the dynamic and seasonally influenced circulation patterns of respiratory pathogens during the study period.

## 4 Discussion

The high prevalence of co-infections observed in this study is consistent with recent multi-center pediatric surveillance data demonstrating that co-infection rates can exceed 25%–30% during peak respiratory virus seasons (32). Several studies have shown that children with viral–bacterial co-infections, particularly involving *M. pneumoniae* or RSV, are more likely to experience prolonged fever, increased hospitalization rates, and greater healthcare utilization compared with mono-infected patients (33). These findings underscore the clinical importance of multiplex diagnostic approaches in pediatric settings.

Furthermore, the age-dependent distribution patterns observed in this study highlight the necessity of age-tailored prevention strategies. Evidence from southern China suggests approximately 30%–40% of children aged 5–15 years have *M. pneumoniae* infection, driven by heightened social interactions and exposure within school environments (34). This aligns with findings from the United States showing that nearly 40% of school-aged children and 12% of infants test positive for *M. pneumoniae* during outbreaks (35). The recent introduction and expanded use of RSV monoclonal antibodies and maternal immunization strategies may significantly reduce disease burden in younger age groups if effectively implemented (36). Continued post licensure surveillance will be essential to evaluate the population-level impact of these interventions. In addition, the seasonal patterns of respiratory pathogens demonstrate significant variability, particularly with Influenza A infection, which peaked with 11,311 reported cases (children with Influenza A infection) in November 2023. This pattern aligns with typical influenza trends where the winter months see an upsurge in incidence rates. From a public health perspective, the pronounced seasonal overlap among influenza viruses, RSV, rhinovirus, and SARS-CoV-2 raises concerns regarding concurrent outbreaks and healthcare system strain. Consequently, integrated surveillance systems that simultaneously monitor multiple respiratory pathogens are increasingly recommended to support early warning mechanisms and timely intervention strategies (37). These findings emphasize the importance of enhancing surveillance systems that track co-infections to inform treatment protocols and optimize resource allocation.

The need to strengthen antimicrobial stewardship (AMS) is a critical lesson underscored by surveillance data; however, the ability to act on these insights varies significantly between high-income countries and low- and middle-income countries (LMICs). The World Health Organization estimates that more than 50% of antibiotics prescribed for children with acute respiratory infections in LMIC pediatric settings are unnecessary, largely due to diagnostic uncertainty and limited access to rapid pathogen testing (38). Viral URTI account for the majority of childhood respiratory illnesses; however, empirical antibiotic use persists, contributing substantially to antimicrobial resistance (AMR). In well-resourced settings, established AMS programs have demonstrated effectiveness in optimizing antibiotic use and enhancing patient outcomes (39, 40). These initiatives are typically supported by robust laboratory infrastructure, broad access to rapid molecular diagnostics, and dedicated multidisciplinary teams, including infectious disease specialists and pharmacists who conduct prospective audit and feedback activities (41). Nevertheless, such resource-intensive models are often not directly transferable to LMICs, where essential prerequisites for their successful implementation are frequently lacking (41). The challenges in these regions are multifaceted, encompassing a severe shortage of trained healthcare personnel, limited availability of essential diagnostic tools, and fragmented supply chains. These systemic constraints hinder effective execution of AMS interventions and contribute to the continued prevalence of inappropriate antibiotic use (41).

Ongoing strategies to address this challenge emphasize the integration of antimicrobial stewardship programs (ASP) into primary and secondary healthcare systems. Evidence from multi-center studies in Asia and sub-Saharan Africa demonstrates that stewardship interventions combining clinician education, guideline dissemination, and audit-feedback mechanisms can reduce inappropriate antibiotic prescriptions for children with pediatric URTI by 20%–40% without adversely affecting clinical outcomes (42, 43). These findings underscore the importance of structured stewardship frameworks even in resource-limited settings. The expansion of rapid and affordable diagnostic tools represents another critical strategy for improving URTI management. Point-of-care molecular assays and host-response biomarkers, such as C-reactive protein and procalcitonin, have shown promise in distinguishing viral from bacterial infections and guiding antibiotic decision-making in LMIC outpatient settings (44). At the policy level, national action plans on AMR increasingly highlight the role of standardized clinical pathways and community-based interventions. Public education campaigns aimed at caregivers, coupled with regulatory measures restricting over-the-counter antibiotic sales, have been associated with measurable reductions in antibiotic misuse for childhood respiratory infections in several middle-income countries (45). Strengthening surveillance networks and linking pathogen epidemiology with prescribing data may further enhance rational antibiotic use.

The epidemiological patterns identified in this study are highly relevant to low-and middle-income countries (LMIC), where respiratory tract infections in children remain a leading cause of outpatient visits, hospitalizations, and preventable mortality. In many LMIC settings, viral pathogens such as RSV, influenza, and rhinovirus account for the majority of upper respiratory tract infections in children, yet diagnostic capacity remains limited, resulting in substantial under-recognition of co-infections and seasonal trends (46). Recent multi-county surveillance studies from Southeast Asia and sub-Saharan Africa demonstrate that *M. pneumoniae* and RSV are frequently under-diagnosed contributors to pediatric respiratory disease, particularly among school-aged children and infants, respectively (47). In LMIC, inappropriate antibiotic prescribing for pediatric upper respiratory tract infections remains a persistent challenge. Large observational studies indicate that antibiotics are prescribed in over half of pediatric URTI visits, despite viral etiologies predominating (48, 49). The lack of rapid diagnostic tools and limited access to laboratory-based PCR testing are major drivers of empirical antibiotic use, contributing to the acceleration of antimicrobial resistance. Evidence from randomized trials conducted in Vietnam and sub-Saharan Africa shows that the introduction of point-of-care molecular diagnostics and biomarker-guided treatment algorithms can significantly reduce unnecessary antibiotic exposure without increasing adverse outcomes (50).

From a public health perspective, strengthening integrated respiratory pathogen surveillance systems in LMIC is essential for early outbreak detection, rational resource allocation, and vaccine policy planning (51). Sentinel surveillance networks established in middle-income countries such as China, Brazil, and South Africa have demonstrated that linking laboratory-confirmed pathogen data with age- and season-specific trends can substantially improve epidemic preparedness and response (52, 53). The comprehensive surveillance approach employed in the present study may therefore serve as a scalable model for LMIC healthcare systems seeking to optimize pediatric respiratory infection management under constrained resources (51, 54). Collectively, these ongoing strategies—encompassing stewardship programs, diagnostic innovation, clinician and community education, and supportive health policies—are essential to improving URTI management and mitigating AMR risks in LMIC (55, 56). Integrating such approaches with comprehensive respiratory pathogen surveillance, as demonstrated in the present study, may provide a scalable model for evidence-based pediatric respiratory infection management in diverse healthcare settings.

This study has several strengths. It used a large, diverse sample of 270,526 children with upper respiratory symptoms, enhancing reliability and generalization. The robust methodology, including multiplex PCR testing for multiple pathogens, enabled detailed analysis of single and co-infections. An age-stratified approach provided insights into pathogen prevalence across developmental stages. Conducted in the context of post-pandemic public health challenges, the findings are relevant to current trends and healthcare responses. Lastly, the results highlight the importance of co-infection dynamics for clinical and public health strategies. However, the absence of historical data from per and during the peak COVID-19 could be the limitation of this study, and the retrospective design may introduce biases and fail to capture all clinically relevant variables. Variations in testing protocols or laboratory capabilities could affect result consistency. Data from a single hospital may limit generalization. Focusing on upper respiratory pathogens might overlook lower respiratory infections. Finally, while participants were categorized by age, other potential confounding factors (e.g., health conditions, socioeconomic status) were not accounted for.

## 5 Conclusion

In summary, the findings of this study elucidate the intricate interplay of respiratory pathogens across different age groups of children and seasonal cycles. These insights are critical for developing effective public health interventions aimed at mitigating the burden of respiratory illnesses in children. By implementing targeted vaccination strategies, enhancing surveillance systems, and fostering public education, healthcare systems can improve their response to respiratory pathogens, ultimately safeguarding public health and reducing morbidity and mortality in children associated with these infections.

## Data Availability

The raw data supporting the conclusions of this article will be made available by the authors, without undue reservation.
